# MVA Vectors Expressing Conserved Influenza Proteins Protect Mice against Lethal Challenge with H5N1, H9N2 and H7N1 Viruses

**DOI:** 10.1371/journal.pone.0088340

**Published:** 2014-02-11

**Authors:** Annett Hessel, Helga Savidis-Dacho, Sogue Coulibaly, Daniel Portsmouth, Thomas R. Kreil, Brian A. Crowe, Michael G. Schwendinger, Andreas Pilz, P. Noel Barrett, Falko G. Falkner, Birgit Schäfer

**Affiliations:** 1 Vaccine R&D, Baxter Bioscience, Orth/Donau, Austria; 2 Pharmacology, Baxter Bioscience, Orth/Donau, Austria; 3 Global Pathogen Safety, Baxter Bioscience, Vienna, Austria; 4 Biologicals R&D, Baxter Bioscience, Orth/Donau, Austria; Public Health Agency of Canada, Canada

## Abstract

**Background:**

The availability of a universal influenza vaccine able to induce broad cross-reactive immune responses against diverse influenza viruses would provide an alternative to currently available strain-specific vaccines. We evaluated the ability of vectors based on modified vaccinia virus Ankara (MVA) expressing conserved influenza proteins to protect mice against lethal challenge with multiple influenza subtypes.

**Methods:**

Mice were immunized with MVA vectors expressing H5N1-derived nucleoprotein (NP), the stem region of hemagglutinin (HA), matrix proteins 1 and 2 (M1 and M2), the viral polymerase basic protein 1 (PB1), or the HA stem fused to a quadrivalent matrix protein 2 extracellular domain (M2e). Immunized mice were challenged with lethal doses of H5N1, H7N1 or H9N2 virus and monitored for disease symptoms and weight loss. To investigate the influence of previous exposure to influenza virus on protective immune responses induced by conserved influenza proteins, mice were infected with pandemic H1N1 virus (H1N1pdm09) prior to immunization and subsequently challenged with H5N1 virus. Antibody and T cell responses were assessed by ELISA and flow cytometry, respectively.

**Results:**

MVA vectors expressing NP alone, or co-expressed with other conserved influenza proteins, protected mice against lethal challenge with H5N1, H7N1 or H9N2 virus. Pre-exposure to H1N1pdm09 increased protective efficacy against lethal H5N1 challenge. None of the other conserved influenza proteins provided significant levels of protection against lethal challenge. NP-expressing vectors induced high numbers of influenza-specific CD4^+^ and CD8^+^ T cells and high titer influenza-specific antibody responses. Higher influenza-specific CD4^+^ T cell responses and NP-specific CD8^+^ T cell responses were associated with increased protective efficacy.

**Conclusions:**

MVA vectors expressing influenza NP protect mice against lethal challenge with H5N1, H7N1 and H9N2 viruses by a mechanism involving influenza-specific CD4^+^ and CD8^+^ T cell responses.

## Introduction

Influenza viruses of subtype A to which the human population has not previously been exposed have the potential to cause pandemic disease resulting in substantial morbidity and mortality. The greatest threat to global human health is considered that posed by a number of avian influenza viruses expressing hemagglutinin (HA) subtypes H5, H7 and H9 [1], to which the human population is essentially naive.

Vaccination is the most effective intervention to prevent influenza virus infection [2]. Most currently available inactivated influenza vaccines are formulated to include the two major viral surface proteins, HA and neuraminidase (NA), that induce antibody responses that protect against antigenically matched or closely related viruses [3], [4]. However, because HA and NA antibody responses are largely subtype-specific, conventional influenza vaccines do not provide efficacious heterosubtypic immunity. Moreover, considerable antigenic variation occurs within HA and NA subtypes, and vaccine effectiveness may be limited against antigenically drifted strains. Since the manufacture of vaccines which are antigenically matched to a novel influenza virus can take several months, there is a concern that vaccine shortfalls could occur in the event of a pandemic caused by a novel, highly pathogenic influenza virus. One major goal of global pandemic preparedness plans is therefore the development of “universal” vaccines which have the potential to protect not only against currently circulating influenza virus strains but also against any strain or subtype which may emerge in the future [5].

Several approaches have been taken towards the development of universal influenza vaccines (reviewed in [6], [7]). The recent discovery of several naturally-occurring human antibodies against antigenically-conserved epitopes in the stem region of the HA protein [8]–[10], which cross-neutralize and protect animals from challenge with a broad range of influenza viruses, raised the possibility that it might be possible to design a vaccine capable of inducing protective levels of such broadly cross-reactive antibodies. In animal models it has been reported that immunization with the HA stem (“headless HA”) can induce antibody responses capable of protecting mice from lethal virus challenge [11]–[13].

A second approach with the potential to induce broad heterosubtypic immunity involves immunization with antigenically-conserved influenza proteins [14], [15] such as nucleoprotein (NP), matrix protein 1 and 2 (M1 and M2) [16]–[19], and the extracellular domain of the matrix protein 2 (M2e) [20], [21]. Delivery of individual or combinations of these proteins can be achieved by the use of viral vectors, such as modified vaccinia virus Ankara (MVA). Vectors based on MVA do not replicate in human cells but induce highly potent antigen-specific CD4^+^ and CD8^+^ T cell responses as well as high antigen-specific antibody titers [22], [23]. MVA induces dendritic cell (DC) activation, involving the upregulation of molecules involved in antigen uptake, cytokines and cytokine receptors, substantially increasing the ability of DCs to act as antigen presenting cells [24]. MVA vectors are therefore described as having the ability to “self-adjuvant”. MVA vectors expressing various combinations of NP, M1, HA and NA have been evaluated in animal models [25]–[34], and phase I clinical studies of an MVA vector expressing NP and M1 indicate that this approach might be efficacious to prevent influenza infection in humans [35], [36]. It is thought that robust T cell responses induced by MVA expressing NP and M1 are at least partly facilitated by boosting of pre-existing immune responses due to previous influenza infection. In non-clinical studies, infection of animals with non-lethal doses of influenza virus has also been shown to prime for more robust immune responses to conserved influenza proteins [37], [38]. MVA vectors have been safely administered to thousands of individuals, including those with compromised immune systems [22], [23], [39].

In the present study, we evaluated the ability of MVA vectors expressing various combinations of M1, M2, PB1, NP, a quadrivalent M2e fusion protein, or the stem region of HA to protect mice from lethal virus challenge with H5N1, H7N1 and H9N2 viruses. The role of CD4^+^ and CD8^+^ T cell responses in protection against lethal challenge was evaluated. To investigate the possibility that heterosubtypic immune responses induced by previous influenza infections might be boosted by MVA-expressed conserved influenza proteins, additional studies were done in mice which had previously been exposed to the 2009 pandemic H1N1pdm09 virus.

## Materials and Methods

### Ethics Statement

All animal experiments were reviewed by the Baxter Bioscience Institutional Animal Care and Use Committee (IACUC Vienna/Orth) and approved by internal animal welfare officers. Animal experiments were conducted in accordance with Austrian laws on animal experimentation and approved by Austrian regulatory authorities (permit number LF1 TVG-38/009-2011). Experiments were conducted according to guidelines set out by the Association for Assessment and Accreditation of Laboratory Animal Care International (AAALAC). Animals were housed according to EU guidelines, in housing facilities accredited by the AAALAC, and monitored daily. For blood sampling and virus challenge, all animals were anaesthetized with isoflurane using a UNO – Univentor Anaesthesia Unit according to the manufacturer’s protocol. Humane endpoints were used during the study, based on weight loss – animals with >25% weight loss were sacrificed using carbon dioxide treatment. All efforts were made to minimize suffering.

### Viruses and Vaccines

Influenza H5N1 strain A/Vietnam/1203/2004 and H1N1pdm09 strain A/California/07/2009 were obtained from Centers for Disease Control and Prevention (Atlanta, USA). The H7N1 A/chicken/Rostock/45/1934 was obtained from the department of Global Pathogen Safety, Baxter, Vienna. Mouse-adapted H9N2 strain A/chicken/Hong Kong/G9/97 was previously described [40]. MVA 1974/NIH clone 1 was kindly provided by B. Moss (National Institutes of Health).

### Generation of Recombinant MVA Vectors

DNA sequences encoding conserved influenza proteins based on H5N1/Vietnam/1203/2004 (M1, M2, PB1, HA stem (hlHA) and HA stem/M2e) DNA sequences were chemically synthesized (Geneart, Regensburg, Germany). In the HA stem sequence, the HA1 sequence between the two highly-conserved cysteines 58 and 290, which forms the globular head of intact HA1, was replaced by a linker consisting of four glycines, as previously described [13]. The quadrivalent M2e fusion protein contains M2e sequences derived from H5N1/Vietnam/1203/2004, H9N2/chicken/Korea/SH0913/2009, H7N2/New York/107/2003 and H1N1/New York/3315/2009, linked to the 5′– and 3′– end of the HA1 sequence by a linker of three glycines (GGG) and to each other by the amino acid linker GSAGSA.

M2, PB1, HA stem or HA stem/M2e sequences were cloned downstream of the vaccinia early/late promoter mH5 [41] in plasmid pDM-D4R [42], resulting in plasmids pDM-M2-VN, pDM-PB1-VN, pDM-hlHA and pDM-hlHA/M2e, respectively. M1 was cloned downstream of the strong vaccinia early/late promoter selP in plasmid pDM-D4R, resulting in plasmid pDM-M1-VN. These plasmids were used to recombine the M1, M2, PB1, HA stem or HA stem/M2e expression cassettes into the MVA D4R/D5R region as previously described [42], resulting in vectors MVA-PB1, MVA-M1, MVA-M2, MVA-hlHA (expressing the HA stem, “headless HA” [hlHA]), and MVA-hlHA/M2e, respectively.

An expression cassette containing H5N1/Vietnam/1203/2004 NP was excised from plasmid pDD4-mH5-mNP-VN [43] and cloned into plasmid pd3-lacZ-gpt [44], resulting in plasmid pd3-lacZ-mH5-NP-VN. The NP expression cassette was recombined into the MVA deletion III (del III) locus by infection of chicken embryo fibroblasts (CEF) with MVA, co-transfection with plasmid pd3-lacZ-mH5-NP-VN, and selection using the transient marker stabilization method [45], resulting in vector MVA-NP.

For the construction of vectors co-expressing NP in addition to either HA stem or HA stem/M2e, CEF were infected with MVA-hlHA or MVA-hlHA/Me2 and subsequently co-transfected with plasmid pd3-lacZ-mH5-NP-VN. The resulting vectors, MVA-hlHA-NP and MVA-hlHA/M2e-NP, contain the HA stem or HA stem/M2e expression cassette in the D4R/D5R region, and the NP expression cassette in the del III locus. Cloning was verified by PCR analysis as previously described [28]. Recombinant MVA vectors propagated in CEF were purified by sucrose cushion, as previously described [46]. Vector titers were determined by plaque titration in DF-1 cells or TCID_50_ assay on CEF cells as previously described [28], [47].

### Determination of Virus Challenge Doses in Balb/c Mice

Groups of 10 female Balb/c mice were challenged intranasally with 50 µl of 10-fold serial dilutions (in PBS) of virus stock, or PBS as control. Infected mice were monitored for weight loss for 14 days. The lethal dose fifty percent (LD_50_), that is, the virus dose at which 50% of mice survived lethal challenge, was calculated using the Kundi-Logit software [48].

### Immunization and Challenge of Mice

Groups of six female Balb/c mice (11 to 12 weeks old) were immunized intramuscularly (i.m.) twice, three weeks apart, with 10^6^ pfu recombinant MVA vectors or wild-type (wt) MVA vector as control. In experiments where mice were primed by exposure to pandemic H1N1 before vaccination, mice were infected intranasally, six weeks prior to immunization, with 100 TCID_50_ of H1N1pdm09. Blood samples for ELISA assays were obtained three weeks after immunization. Mice were challenged intranasally three weeks after the second immunization with 10^3^ TCID_50_ (42 LD_50_) H5N1, 2.5×10^4^ TCID_50_ (32 LD_50_) H9N2 or 1.7×10^4^ TCID_50_ (16 LD_50_) H7N1, and weight loss and symptoms were monitored for 14 days. Ruffled fur, curved posture, apathy and death were scored as 1, 2, 3 and 4, respectively. For the evaluation of T cell responses, spleens were collected seven days after the second immunization.

### Detection of Antibodies and T Cells by ELISA and Flow Cytometry

Antibody responses to whole H5N1 virus was done by ELISA as described previously [47]. Flow cytometric intracellular IFN-γ staining was used to determine frequencies of hemagglutinin-specific CD4^+^ and CD8^+^ T cells essentially as previously described [28]. Briefly, single-cell suspensions of splenocytes were stimulated overnight in the presence of 10 µg/ml brefeldin A (Sigma) with 3 µg HA/ml inactivated H5N1, H1N1 or H9N2 whole-virus antigen, or with 2 µg/ml peptides from HA (HA 518–526) or NP (NP 147–155). Cells were then incubated with LIVE/Dead Violet Kit (VIVID, Molecular Probes), stained with rat anti-mouse CD4-APC and CD8-APC H7 antibodies and, after permeabilization with 0.08% saponin (Sigma), with rat anti-mouse IFN-γ FITC and CD3-PerCP antibodies (all BD Biosciences). At least 100,000 viable cells were acquired on a FACSCanto-2 (BD Biosciences), and analyzed using FlowJo software (Tree Star Inc.). Percentages of IFN-γ producing T cells were calculated after gating on VIVID negative, CD3^+^, CD4^+^ or CD8^+^ lymphocytes.

### Statistical Analysis

Statistical analyses were done using GraphPad Prism software (version 5.04). For statistical comparison of clinical symptoms and weight loss data one-way ANOVA and the Tukey-Kramer posttest were used. Statistical differences between survival rates were analyzed using the Kaplan Meyer log rank test. Statistical differences between antibody and T cell responses were calculated using two-way ANOVA and Bonferroni posttests.

## Results

### Generation of MVA Vectors Expressing Conserved Influenza Proteins

**Figure 1:**
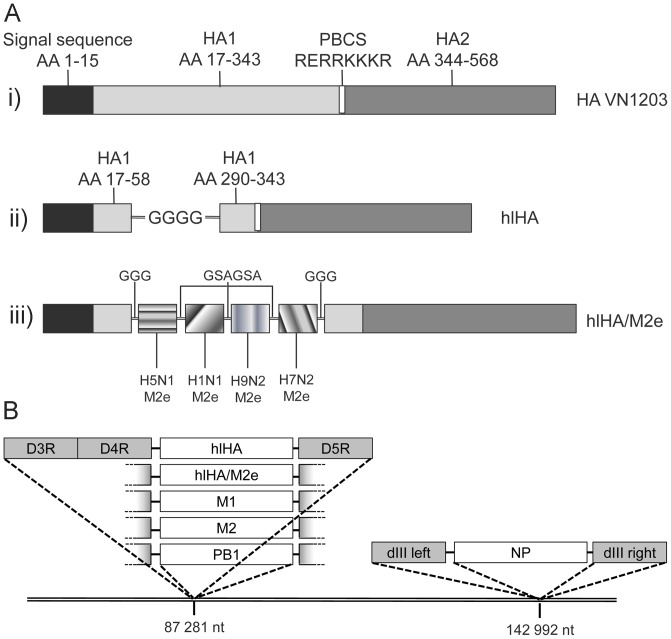
Scheme of HA stem and HA stem/M2e sequence. (**A**) Schematic representation of the HA stem (hlHA) and HA stem/M2e constructs, depicting i) the hemagglutinin (HA) of A/Vietnam/1203/2004 H5N1 (VN1203) from which the hlHA and hlHA/M2e constructs were derived; ii) the HA stem (hlHA) construct which contains the HA signal sequence, the HA1 amino acids (AA) 17–58 and 290–343, which are linked by four glycine (G) residues, the polybasic cleavage site, and the HA2 sequence; iii) the HA stem/M2e fusion protein which contains the M2e domains of H5N1, H1N1, H9N2, H7N2, linked to each other by a GSAGSA linker, and linked to the HA1 sequences by three glycine (G) residues. (**B**) Schematic representation depicting the nucleotide (nt) positions within the MVA vector into which conserved influenza proteins are inserted.

To assess the potential of MVA vectors expressing conserved influenza proteins to induce immune responses capable of protecting against diverse influenza subtypes, a panel of vectors expressing either H5N1-derived NP, PB1, M1, M2, or HA stem (hlHA) were generated (Figure 1). In addition, to investigate the ability of M2e to potentiate the protective effect of the HA stem, an MVA vector was generated which expresses the HA stem fused to four M2e domains derived from H5N1, H9N2, H7N2 and H1N1 (Table 1). To evaluate potential synergistic effects of co-expression of NP and HA stem and/or M2e, vectors were constructed which express both NP and either the HA stem or the HA stem-M2e fusion protein (Figure 1). All vectors were propagated on CEF, achieving titers of 2.6×10^9^ to 2.6×10^10^ pfu/ml after sucrose purification. All MVA-expressed influenza proteins, except PB1, were detected by western blot in infected CEF lysates; for PB1, the correct sequence and orientation of the expression cassette within vector MVA-PB1 was confirmed by sequencing (data not shown).

**Table 1:** Conserved M2e sequences used to generate quadrivalent M2e coding sequence.

Virussubtype	Virus^a^	Amino acid sequence^b^
H5N1	A/Viet Nam/1203/2004	MSLLTEVETPTRNEWECRCSDSSD
H1N1^c^	A/New York/3315/2009	MSLLTEVETP**I**RNEW**G**CRC**N**DSSD
H7N2	A/New York/107/2003	MSLLTEVETP**I**R**KG**WEC**N**CSDSSD
H9N2	A/chicken/Korea/SH0913/2009	MSLLTEVETPTRN**G**WEC**K**CSDSSD

a Example of a specific virus strain coding the respective M2e sequence.

b Amino acid sequence of the respective M2e. Differences to the H5N1 M2e sequence are highlighted in bold.

C Amino acid sequence of H1N1 is identical to that of H2N2 A/Korea/426/68 and H3N2 A/NewYork/392/2004.

### Protective Efficacy of MVA-expressed Conserved Influenza Proteins in Mice

The protective efficacy of MVA vectors expressing conserved influenza proteins was assessed by immunizing groups of Balb/c mice twice, three weeks apart, with 10^6^ pfu of recombinant MVA vectors, and challenge, three weeks later, with lethal doses of wild-type H5N1 (42 LD_50_), wild-type H7N1 (16 LD_50_) or mouse-adapted H9N2 (32 LD_50_). The numbers of immunized mice in each group surviving two weeks after virus challenge are shown in Table 2.

**Table 2:** Protective efficacy of MVA vectors in Balb/c mice.

*MVA Vector*	*Expressed influenza protein(s)*	Challenge Virus			
		H5N1	H5N1, primed^a^	H9N2	H7N1
MVA-NP	H5N1 NP	6/12 (50)	12/12 (100)	6/6 (100)	11/12 (92)
MVA-hlHA-NP	H5N1 HA stem, H5N1 Nucleoprotein	6/12 (50)	12/12 (100)	12/12 (100)	12/12 (100)
MVA-hlHA/M2e-NP	H5N1 HA stem, M2e fusion protein, H5N1 NP	9/12 (75)	11/12 (92)	12/12 (100)	12/12 (100)
MVA-hlHA	H5N1 HA stem	2/12 (17)	9/12 (75)	1/6 (17)	1/12 (8)
MVA-hlHA/M2e	H5N1 HA stem, M2e fusion protein	1/12 (8)	9/12 (75)	2/6 (33)	1/12 (8)
MVA-M1	H5N1 Matrix protein 1	1/6 (17)	5/12 (42)	0/6 (0)	3/12 (25)
MVA-M2	H5N1 Matrix protein 2	4/12 (33)	8/12 (67)	3/6 (50)	0/12 (0)
MVA-PB1	H5N1 Polymerase basic protein 1	2/12 (17)	8/12 (67)	1/6 (17)	2/12 (17)
wtMVA	none	3/18 (17)	11/18 (61)	5/12 (42)	0/12 (0)
PBS	none	6/18 (33)	5/18 (28)	2/12 (17)	0/12 (0)

Data are n/N (%) protected mice, 14 days after challenge with with 42 LD_50_ of H5N1 VN1203, 32 LD_50_ of mouse-adapted H9N2 HK/G9 or 16 LD_50_ of H7N1 RO/34.

a mice were immunologically primed by infecting with H1N1 six weeks prior to immunization.

Vectors which expressed NP, either alone or in combination with the HA stem or with the HA stem and M2e fusion protein, protected mice against lethal challenge to an extent which was substantially and significantly above those seen in animals which received wtMVA or PBS control. MVA-NP protected 50% of animals against lethal challenge with wild-type H5N1 (p = 0.1479), 100% of animals against H9N2 challenge (p = 0.002) and 92% of animals against H7N1 challenge (p<0.0001). MVA-hlHA-NP protected 50% of animals against lethal challenge with wild-type H5N1 (p = 0.125), 100% of animals against H9N2 challenge (p = 0.002) and 100% of animals against H7N1 challenge (p<0.0001). MVA-hlHA/M2e-NP protected 75% of animals against lethal challenge with wild-type H5N1 (p = 0.0057), 100% of animals against H9N2 challenge (p = 0.002) and 100% of animals against H7N1 challenge (p<0.0001). In animals receiving wtMVA, the total number of survivors was 3/18 (17%) against H5N1 challenge, 5/12 (42%) against H9N2 challenge and 0/12 (0%) against H7N1 challenge. In mice receiving PBS control, the total number of survivors was 6/18 (33%) against H5N1 challenge, 2/12 (17%) against H9N2 challenge and 0/12 (0%) against H7N1 challenge.

There was no statistically significant protective effect of wtMVA compared to PBS control with respect to protection against lethal challenge against H5N1 (p = 0.4854), H7N1 (p = 0.8583) or H9N2 (p = 0.5215). Vectors expressing only M1, M2, PB1, HA stem or HA stem/M2e did not induce significantly higher levels of protection compared to wtMVA or PBS control against lethal challenge with either H5N1, H9N2 or H7N1 (Table 2). In some experiments animals receiving MVA expressing M1 alone had a lower rate of survival than animals receiving wtMVA or PBS control. However, the differences between survival rates among the different groups was not statistically significant, with the exception of a slightly significantly higher survival rate against lethal challenge with H5N1 (p = 0.0172) for animals receiving PBS control compared to animals receiving MVA-M1.

To investigate the potential of recombinant MVA vectors to boost immune responses induced by previous infection with a heterologous influenza virus, mice were primed by infection with a non-lethal dose (100 TCID_50_) of H1N1pdm09 virus, six weeks prior to immunization with MVA vectors, and subsequent challenge with 42 LD_50_ wild-type H5N1. Pre-exposure of animals to H1N1pdm09 increased the ability of NP-expressing vectors to protect against challenge with H5N1, such that protection against lethal challenge was significant or borderline significant compared to animals receiving wtMVA or PBS control. Animals immunized with MVA-NP, MVA-hlHA-NP and MVA-hlHA/M2e-NP which had been previously exposed to H1N1pdm09 were 100% (p = 0.0169), 100% (p = 0.0169) and 92% (p = 0.0781) protected against H5N1 challenge, respectively; only 11/18 (61%) of H1N1pdm09-primed mice which received wtMVA and 5/18 (28%) of mice receiving PBS control were protected against H5N1 challenge. No significantly higher levels of protection against lethal challenge, compared to wtMVA or PBS control, was provided by vectors expressing only M1, M2, PB1, HA stem or HA stem/M2e (Table 2).

### Prevention of Weight Loss and Disease Symptoms

**Figure 2:**
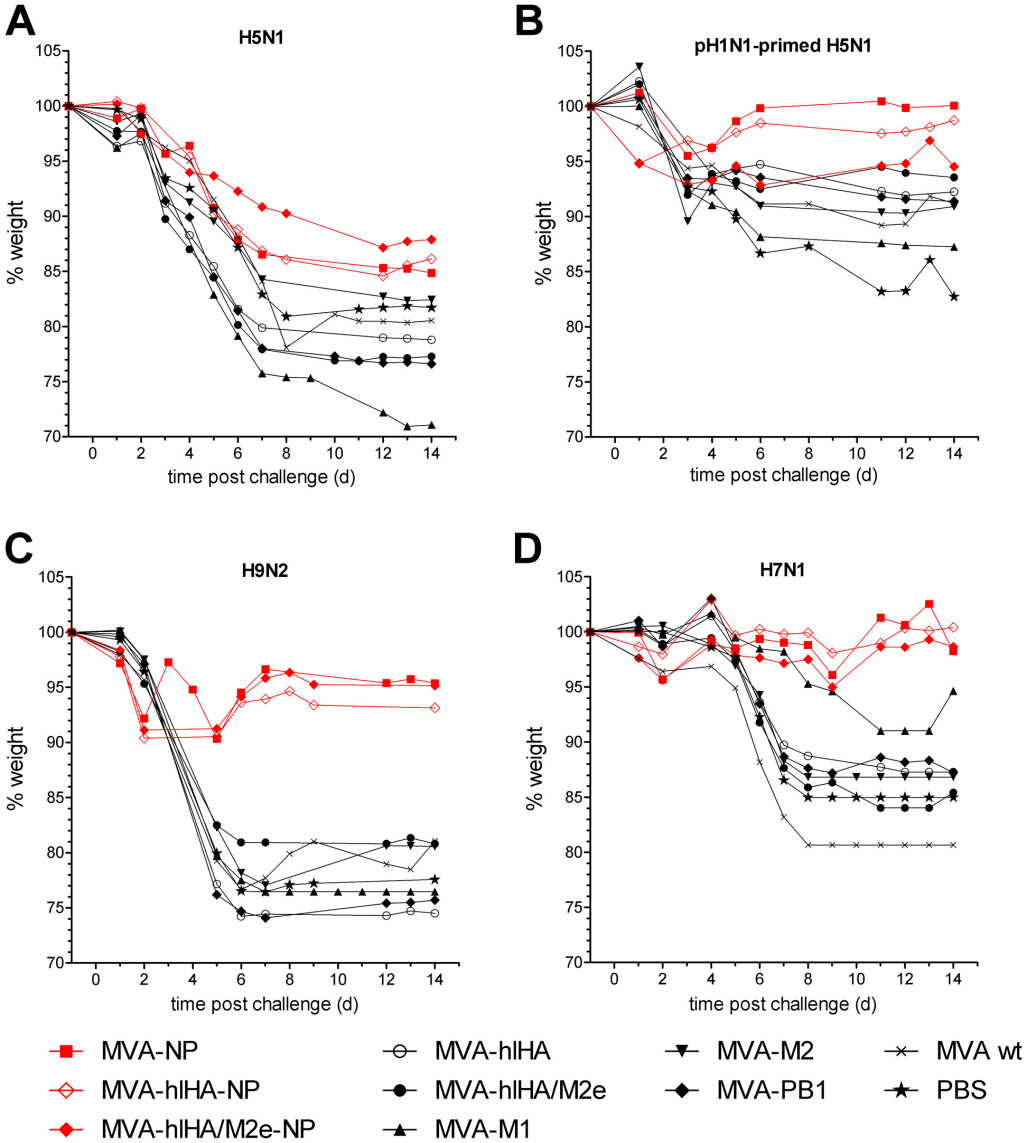
Weight loss after H5N1, H9N2 and H7N1 challenge. Mice were immunized twice, three weeks apart, with MVA vectors and challenged three weeks later with (**A and B**) 42 LD_50_ of H5N1 VN1203, (**C**) 32 LD_50_ of mouse-adapted H9N2 HK/G9 or (**D**) 16 LD_50_ of H7N1 RO/34. (**B**) Primed mice were infected intranasally with 100 TCID_50_ H1N1pdm09 virus, six weeks before immunization. Animals were monitored for 14 days after challenge. Shown are the daily variations in weight, as percentages compared to before virus challenge.

Although protection against lethal challenge with H5N1 was significant for some vectors expressing NP, reductions in weight loss were not significant for any non-primed immunized mice challenged with H5N1, compared to mice receiving wtMVA or PBS control (Figure 2A); however, significant reductions in weight loss were achieved in H5N1-challenged, H1N1-primed mice immunized with MVA-NP (p<0.001) and MVA-hlHA-NP (p<0.01) compared to mice receiving wtMVA or PBS control (Figure 2B). Weight loss as a result of infection with H9N2 or H7N1 viruses was substantially and significantly reduced by immunization with NP-containing MVA vectors compared to mice receiving wtMVA or PBS control. Mice immunized with NP-expressing vectors and challenged with H9N2 (Figure 2C) or H7N1 (Figure 2D) showed only slight weight loss immediately post-challenge, and recovered to pre-challenge or near pre-challenge weights by 14 days post-challenge. The reduction in weight loss was highly significant (p<0.001) for all NP-containing vectors against H7N1 challenge and also significant for H9N2-challenged mice receiving MVA-NP (p<0.01), MVA-hlHA-NP (p<0.05) and MVA-hlHA/M2e-NP (p<0.05). Mice receiving other influenza protein-expressing vectors, wtMVA or PBS control lost weight rapidly after challenge with H5N1, H7N1 or H9N2, and were substantially below pre-challenge weights by the end of the observation period.

**Figure 3:**
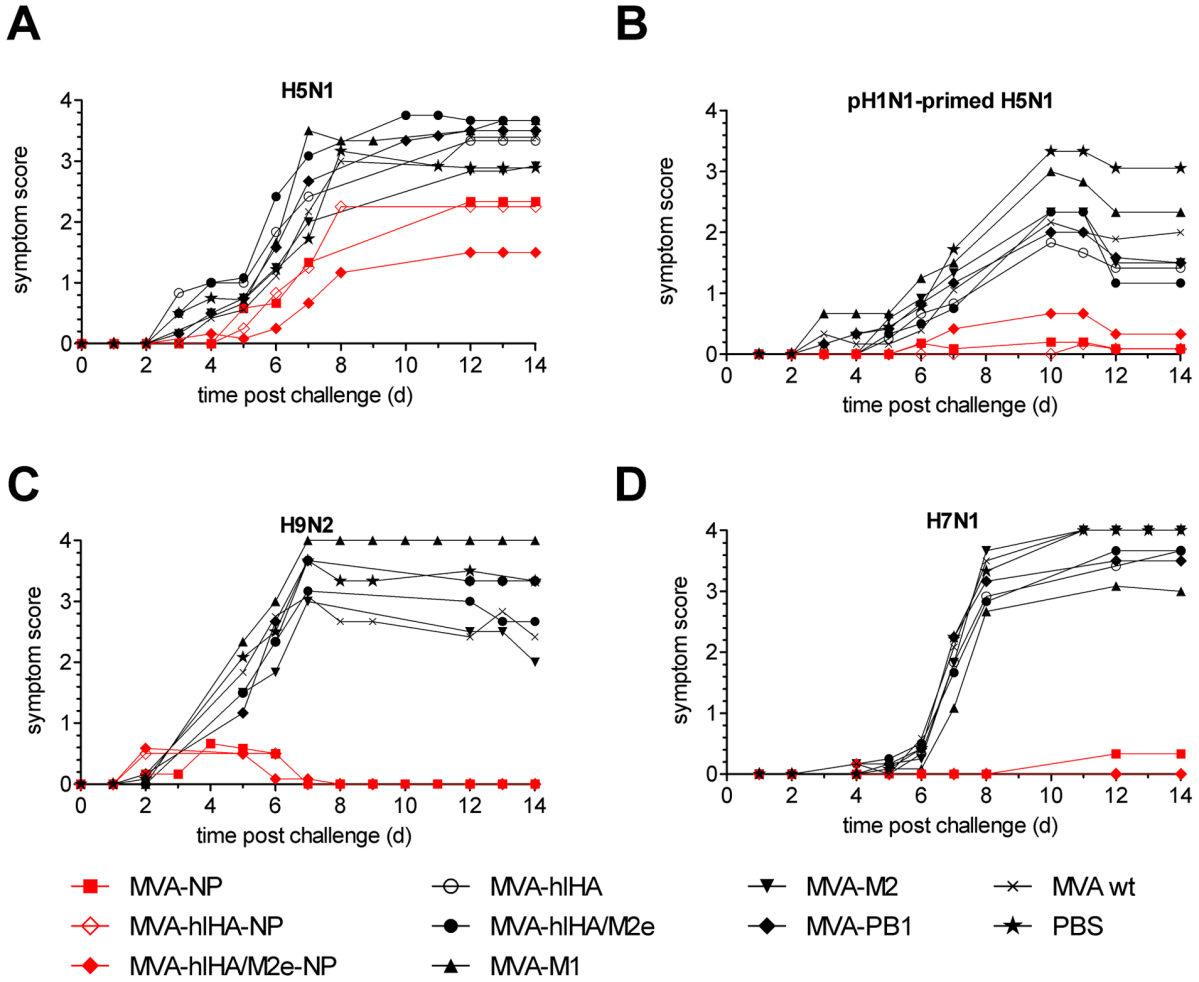
Symptom scores after H5N1, H9N2 and H7N1 challenge. Mice were immunized twice, three weeks apart, with MVA vectors and challenged three weeks later with (**A and B**) 42 LD_50_ of H5N1 VN1203, (**C**) 32 LD_50_ of mouse-adapted H9N2 HK/G9 or (**D**) 16 LD_50_ of H7N1 RO/34. (**B**) Primed mice were infected intranasally with 100 TCID_50_ H1N1pdm09 virus, six weeks before immunization. Animals were monitored for 14 days after challenge. Shown are the cumulative mean symptom scores whereby ruffled fur, curved posture, apathy and death were scored as 1, 2, 3 and 4, respectively.

In agreement with the weight loss data, disease symptoms were not significantly reduced in any non-primed mice challenged with H5N1 (Figure 3A); however, significant reductions in disease symptoms were achieved in H1N1-primed mice immunized with any NP-containing vector and challenged with H5N1 (p<0.001) (Figure 3B). Disease symptoms as a result of infection with H9N2 (Figure 3C) or H7N1 (Figure 3D) viruses were also substantially and significantly reduced by immunization with NP-containing MVA vectors. By the end of the observation period in mice immunized with NP-expressing vectors, 36/36 mice challenged with H9N2 and 34/36 mice challenged with H7N1 were symptom-free. The reduction in disease symptoms was highly significant in mice immunized with any NP-containing vector and challenged with H7N1 (p<0.001) as well as for H9N2-challenged mice which had been immunized with MVA-NP (p<0.001), MVA-hlHA-NP (p<0.001) or MVA-hlHA/M2e-NP (p<0.001). Vectors expressing only M1, M2, PB1, HA stem or HA stem/M2e did not provide significant protection against disease symptoms after challenge with H5N1, H7N1 or H9N2, nor in H1N1-primed mice challenged with H5N1.

### Induction of Influenza-specific Antibodies and CD4^+^ and CD8^+^ T Cells

**Figure 4:**
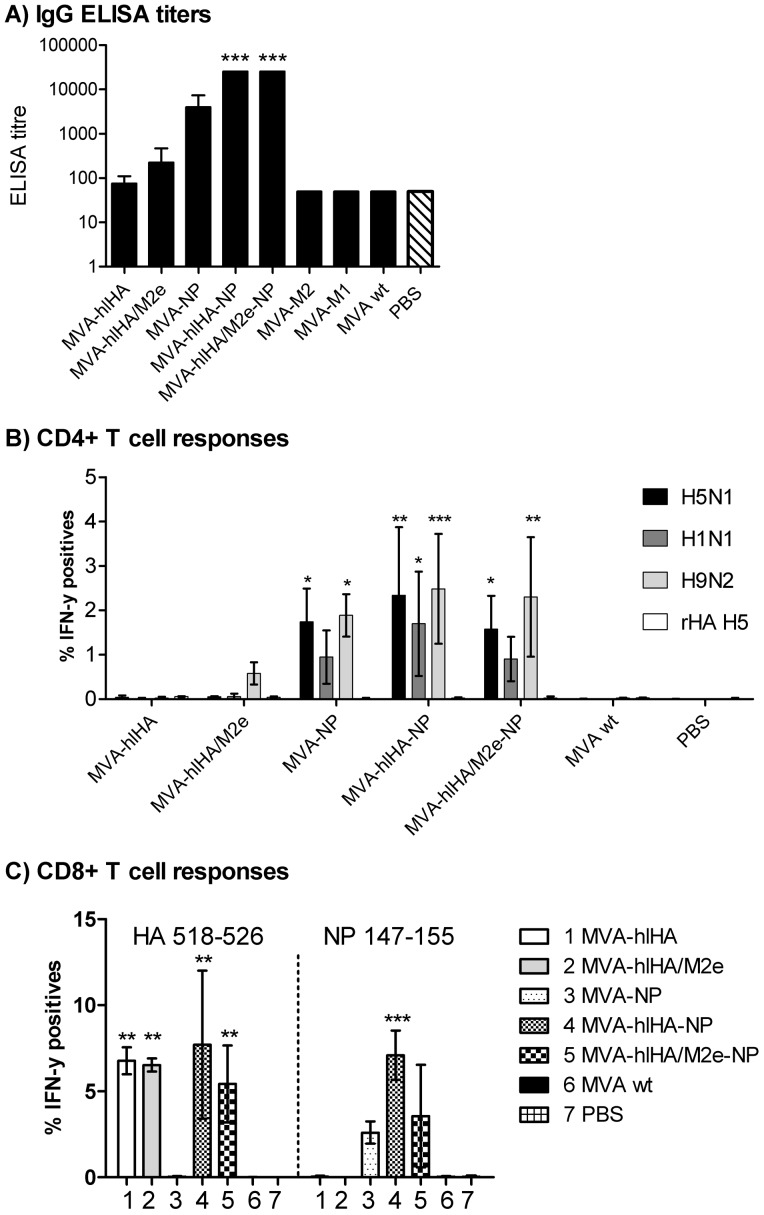
Antibody and T cell responses. (**A**) ELISA titers against whole-virus H5N1. The starting dilution of the assay is 1∶100. (**B**) Percentage of CD4^+^ T cells reacting to whole-virus H5N1, H1N1 and H9N2 and to recombinant H5 hemagglutinin (rHA H5) antigen after immunization of mice with MVA vectors. (**C**) Percentage of CD8^+^ T cells reacting to HA and NP peptides. Asterisks denote statistical significance calculated by two-way ANOVA and Bonferroni posttests (*p<0.01, **p<0.001, ***p<0.0001).

The ability of MVA-expressed conserved influenza proteins to induce influenza-specific antibodies was evaluated by ELISA in sera collected three weeks after the second immunization, using whole H5N1 antigen (Figure 4A). Titers >1000 were induced by MVA-NP, and substantially and significantly higher titers (>10,000, p<0.001) were induced by vectors co-expressing NP and HA stem or NP and HA stem/M2e. In contrast, much lower antibody titers were induced by HA stem or HA stem/M2e in the absence of NP, and no detectable antibodies were found to be induced against M1- or M2-expressing vectors.

To evaluate the ability of MVA-expressed conserved influenza proteins to induce cross-reactive influenza-specific T cell responses, whole-virus antigens from H5N1, H1N1pdm09 and H9N2, as well as recombinant H5 HA (rHA) were used for re-stimulation of splenocytes obtained from immunized mice. High numbers of IFN-γ-expressing CD4^+^ T cells were induced in mice immunized with all NP-expressing vectors, with 1.7 to 2.5% of total CD4^+^ T cells responding to H5N1 and H9N2, and approximately 0.9 to 1.7% responding to pandemic H1N1 (Figure 4B). CD4^+^ T cells responses to H5N1 and H9N2 induced by vectors expressing NP were substantially and significantly above levels observed in mice receiving wtMVA or PBS control. In mice receiving vectors expressing HA stem alone or HA stem/M2e fusion protein, levels of influenza-specific CD4^+^ T cells were not significantly higher than those in mice receiving wtMVA or PBS control.

High frequencies of NP-specific CD8^+^ T cells were also induced by all NP-expressing vectors, with responses ranging from 2.6 to 7.1% of total CD8^+^ T cells (Figure 4C). As expected, no NP-specific CD8^+^ T cells were induced by vectors expressing HA stem alone or HA stem/M2e fusion protein. In contrast, significantly higher (p<0.001) levels of HA-specific CD8^+^ T cell responses were induced by all vectors expressing the HA stem, with responses ranging from 5.4 to 7.7% of total CD8^+^ T cells. No HA-specific CD8^+^ T cells were induced by vectors expressing NP alone.

## Discussion

We evaluated the potential of MVA vectors expressing different combinations of conserved H5N1-derived influenza proteins to protect mice against lethal challenge with a panel of potentially pandemic avian influenza viruses, and investigated the contribution of antibody and T cell responses to protective immunity. MVA vectors expressing NP alone, or in combination with HA stem or with an HA stem/M2e fusion protein, protected mice against lethal challenge with H5N1, H7N1 and H9N2 viruses. Remarkably, H5N1 NP-expressing vectors provided more effective protection against lethal challenge with H7N1 and H9N2 than against H5N1, with 65 of a total of 66 mice (98.5%) immunized with NP-expressing vectors surviving challenge with H7N1 and H9N2. Protective efficacy against H5N1 was lower, at 50 to 75%, likely as a result of the more stringent challenge dose used for H5N1 (42 LD_50_) compared to H7N1 (16 LD_50_) and H9N2 (32 LD_50_). However, in mice which were immunologically primed by previous infection with H1N1pdm09 prior to immunization with NP-expressing vectors, 35 of a total of 36 mice (97%) of mice survived H5N1 challenge. These data support the concept that MVA vectors expressing NP are able to boost pre-existing immune responses due to previous influenza virus infections, such as is found in the general human population. A number of other non-clinical studies have demonstrated the efficacy of NP to protect against influenza, either alone [38], [49]–[52] or in combination with other conserved influenza proteins [25], [36], [53]–[58]. In humans, protection against influenza infection and disease was reported for a small phase I study of an MVA vector expressing NP and M1 [36].

Protection against lethal challenge with H5N1, H7N1 and H9N2 was associated with high CD4^+^ T cell responses against whole-virus antigen but not against recombinant H5 HA, indicating that protection is due to T cell responses against NP but not HA. The association of protection with CD4^+^ T cell responses is in agreement with recent clinical data demonstrating that pre-existing CD4^+^ T cells responding to conserved influenza proteins, including NP, correlate with protection, even in the absence of influenza antibodies [59]. Immunization of mice with NP DNA was also reported to prime CD4^+^ T cells, which contributed to protective immunity [52]. CD4^+^ T cells have been described to have both cytotoxic and anti-viral responses [60]–[62] and to provide cognate help to B-cells [63]. However, our conclusions might be limited by the fact that we did not investigate the CD4^+^ T cell responses against H7N1.

In our study, robust NP-specific CD8^+^ T cell responses were also induced by NP-expressing vectors, thus, NP-specific CD8^+^ T cell responses were also associated with increased protection against lethal challenge. This finding is in agreement with a recent report that CD8+ T cell responses against conserved influenza epitopes correlates with protection against influenza in humans [64]. Robust HA-specific CD8^+^ T cell responses were induced by vectors expressing HA stem, but HA-specific CD8^+^ T cell responses were not associated with protection against lethal challenge or amelioration of disease symptoms in vectors which did not also express NP, despite the fact that the HA stem contains a highly-conserved immunodominant CD8^+^ T cell epitope [65]. In this respect, our data contradict earlier reports that HA stem could induce protective immune responses in mice [11]–[13].

It could be speculated that the reason for the discrepancy between our data and previously reported data with respect to protection against lethal challenge induced by the HA stem might be at least partly due to the higher challenge virus doses which were used in the present study. Whereas previous studies used low challenge doses corresponding to about 1 LD_90_ or 2 LD_50_, [11], [13] we used much higher challenge doses, ranging from 16 to 42 LD_50_. We consider these higher challenge doses to provide a more stringent examination of the protective immune response induced by vaccination. In the present study, protection against lethal challenge with H7N1 and H9N2 viruses was more effective than against challenge with H5N1. However, this might also be due to the less stringent challenge doses used for the H7N1 and H9N2 viruses, due to the lower titer of the virus stocks generated for these viruses. Despite the use of high challenge doses, some mice which received PBS or wtMVA survived challenge with H5N1, H9N2 or H7N1 viruses. There was no statistically significant difference between the number of mice receiving PBS or wtMVA which survived challenge, indicating that there was no protective effect of wtMVA. In some experiments, mice which received MVA vectors expressing M1 only were less well protected than mice receiving either PBS or wtMVA; however the difference in survival among these groups were not statistically significant, with the exception of a slightly significant decrease in survival rates for mice receiving MVA-M1 compared to PBS. These data are thus not indicative of an exacerbating effect of M1 expression in mice challenged with influenza virus.

High titer influenza-specific antibodies (>1000) were induced by the MVA vector expressing NP alone, and even higher titers (>10,000) were induced by vectors expressing both NP and HA stem or HA stem/M2e. Interestingly, much lower antibody titers were induced by HA stem or HA stem/M2e alone, suggesting that the elevated antibody responses were induced by vectors co-expressing NP and HA stem or HA stem/M2e, might be due to a synergistic effect of MVA-expressed NP and HA stem or HA stem/M2e with respect to the induction of influenza-specific antibody. In this respect, a limitation of our study is that the use of whole-virus antigen in the ELISA used for detection of influenza antibodies means that we could not quantify antibody responses induced against individual proteins. As such, we could not determine whether the higher antibody titers were induced by HA or NP, or both. However, irrespective of the specificity of the increased antibody responses, these did not facilitate significantly increased levels of protection against lethal challenge above those achieved by vectors expressing NP alone. It is possible that antibodies induced by NP might have contributed to the protective efficacy of NP-expressing vectors in our study, as NP antibodies have previously been described to specifically promote influenza virus clearance in mice by a mechanism involving both Fc receptors and CD8^+^ T cells, and to facilitate heterosubtypic immunity in B cell-deficient mice [66]. However, other non-clinical studies have reported no protective effect of NP antibodies [67], [68].

No significant protection against lethal challenge or amelioration of weight loss or disease symptoms was provided by vectors expressing M2e (in combination with the HA stem), M1, M2 or PB1. These data are in contrast to some other previous studies which reported protective immune responses induced by these conserved influenza proteins [16]–[20], [69]–[72]. In addition, the immunogenicity and protective efficacy of an MVA vector expressing M1 in addition to NP has been reported [25], [53], [73], including in clinical trials [35], [36]. However, the extent to which M1 contributes to the protective immune responses induced by this vector is not clear. The differences in the conclusions of different studies might be due to a number of factors, including differences in the stringency of the challenge dose, differences in the influenza virus used for challenge, the use of different animal models, the use of proteins derived from different influenza viruses or the use of different methods to deliver the proteins.

In summary, MVA vectors expressing the highly-conserved influenza NP protected mice against lethal challenge with diverse influenza virus subtypes, whereas no other conserved influenza proteins provided significant levels of protection. Protection against lethal challenge mediated by NP-expressing vectors was associated with high CD4+ T cell responses. NP-specific CD8+ T cell responses were also induced by all NP-expressing vectors, suggesting that NP-specific CD8+ T cell responses also play a role in protection of immunized mice against lethal challenge with H5N1, H7N1 and H9N2 viruses. Our data support the concept that MVA vectors expressing NP are able to provide broad heterosubtypic immunity which might be effective in mitigating the severity of a future influenza pandemic. Importantly, robust protection against lethal challenge was demonstrated against avian influenza viruses of HA subtypes H5, H7 and H9, which are considered to pose the greatest risk to global human health. Moreover, the excellent protection provided by NP-expressing MVA vectors against lethal challenge with H7N1 virus suggests that these vectors might also be effective against the novel H7N9 virus which has recently emerged in China [74], [75], hospitalizing hundreds and killing approximately one third of infected individuals.
